# P-1508. *In Vitro* Activity of Aztreonam-avibactam against Enterobacterales Isolates Producing Multiple β-lactamases Collected Globally as a Part of the ATLAS Global Surveillance Program from 2018-2022

**DOI:** 10.1093/ofid/ofae631.1677

**Published:** 2025-01-29

**Authors:** Mark Estabrook, Henry Li, Gregory Stone, Katherine Perez, Daniel F Sahm

**Affiliations:** IHMA, Schaumburg, Illinois; IHMA, Schaumburg, Illinois; Pfizer, Inc., Groton, Connecticut; Pfizer, Inc., Groton, Connecticut; IHMA, Schaumburg, Illinois

## Abstract

**Background:**

Aztreonam-avibactam (ATM-AVI) is a β-lactam/β-lactamase inhibitor combination to treat infections caused by Gram-negative organisms, particularly those carrying metallo-β-lactamases (MBLs) and other β-lactamases. Aztreonam is stable to hydrolysis by MBLs and avibactam inhibits Class A, C, and some Class D enzymes. We examined ATM-AVI activity against Enterobacterales isolates producing one or more β-lactamase collected as a part of the ATLAS global surveillance program (2018-2022).

**Figure:**
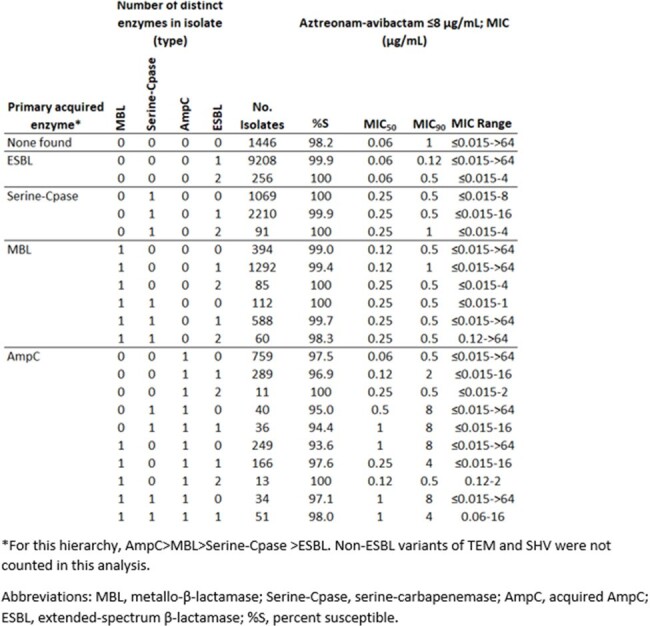

**Methods:**

88,750 isolates from 227 medical centers in 57 countries (excluding mainland China, Canada, and the USA) were collected and tested for susceptibility using the broth microdilution method according to CLSI guidelines. Analysis was performed with CLSI 2024 breakpoints. Isolates testing with meropenem MIC values >1 µg/mL or *Escherichia coli, Klebsiella pneumoniae, K. oxytoca,* or *Proteus mirabilis* isolates testing with ceftazidime and/or aztreonam MIC values >2 µg/mL were screened for β-lactamase genes by PCR, which were sequenced when identified.

**Results:**

One or more extended-spectrum β-lactamase (ESBL), serine-carbapenemase, MBL, or acquired AmpC was identified among 17,013/18,408 isolates characterized. Among isolates carrying one or two ESBLs (51.4%), 99.9-100% tested with ATM-AVI MICs ≤8 µg/mL (MIC_90_ values of 0.12 and 0.5 µg/mL, respectively). Among isolates with a serine-carbapenemase and one or more ESBL (18.3%), 99.9-100% tested with ATM-AVI MICs ≤8 µg/mL (MIC_90_ values of 0.5-1 µg/mL). MBL-positive isolates with or without serine-carbapenemases and/or ESBLs (13.7%) demonstrated MICs ≤8 µg/mL among 98.3-100% of isolates in each category (MIC_90_ values of 0.5-1 µg/mL). Isolates carrying acquired AmpC as well as any combination of additional β-lactamases (9%) tested with ATM-AVI MICs ≤8 µg/mL among 93.6-100% of each population (MIC_90_ values of 0.5-8 µg/mL).

**Conclusion:**

ATM-AVI demonstrated potent *in vitro* activity against Enterobacterales isolates regardless of the number of β-lactamases carried. Slightly reduced potency was observed in populations that carried acquired AmpC. This may be expected as some variants of AmpC with extended-spectrum activity are known to increase ATM-AVI MICs.

**Disclosures:**

**Mark Estabrook, MS**, Pfizer, Inc.: Advisor/Consultant **Henry Li, MS in Biotechnology**, Pfizer, Inc.: Advisor/Consultant **Daniel F. Sahm, PhD**, Pfizer, Inc.: Advisor/Consultant

